# Patterns, Outcomes and Economic Burden of Primary vs. Secondary Bloodstream Infections: A Single Center, Cross-Sectional Study

**DOI:** 10.3390/pathogens13080677

**Published:** 2024-08-09

**Authors:** Ioannis Chandroulis, Georgios Schinas, Anne-Lise de Lastic, Eleni Polyzou, Stamatia Tsoupra, Christos Davoulos, Martha Kolosaka, Vasiliki Niarou, Spyridoula Theodoraki, Dimitrios Ziazias, Foteini Kosmopoulou, Christina-Panagiota Koutsouri, Charalambos Gogos, Karolina Akinosoglou

**Affiliations:** 1 School of Social Sciences, Hellenic Open University, 263 35 Patras, Greece; std528050@ac.eap.gr; 2 School of Medicine, University of Patras, 265 04 Rio, Greece; georg.schinas@gmail.com (G.S.); delastic@gmail.com (A.-L.d.L.); polyzou.el@gmail.com (E.P.); stamtsoupra@gmail.com (S.T.); faykosmopoulou@gmail.com (F.K.); akin@upatras.gr (K.A.); 3 Department of Internal Medicine, University General Hospital of Patras, 265 04 Rio, Greece; chrisda777@gmail.com; 4 Independent Researcher, 272 00 Amaliada, Greece; mkolosaka@gmail.com; 5 Department of Emergency Care, University General Hospital of Patras, 265 04 Rio, Greece; niarouvasiliki@yahoo.com; 6 Department of Internal Medicine, General Hospital of Agrinion, 301 31 Agrinio, Greece; roula-theodor@hotmail.com; 7 Department of Internal Medicine, General Hospital of Nikaia-Pireaus “Agios Panteleimon”, 184 54 Nikaia, Greece; dziazias@gmail.com; 8 Department of Internal Medicine, General Hospital of Patras “Agios Andreas”, 263 32 Patra, Greece; ch.koutsouri@gmail.com; 9 Department of Internal Medicine and Infectious Diseases, Metropolitan General Hospital, 155 62 Athens, Greece; 10 Division of Infectious Diseases, University General Hospital of Patras, 265 04 Rio, Greece

**Keywords:** bloodstream infections, bacteremia, primary bacteremia, secondary bacteremia, clinical outcomes, mortality, hospitalization costs, antibiotic therapy, nosocomial infections, community-acquired infections

## Abstract

Bloodstream infections (BSIs) can be primary or secondary, with significant associated morbidity and mortality. Primary bloodstream infections (BSIs) are defined as infections where no clear infection source is identified, while secondary BSIs originate from a localized infection site. This study aims to compare patterns, outcomes, and medical costs between primary and secondary BSIs and identify associated factors. Conducted at the University Hospital of Patras, Greece, from May 2016 to May 2018, this single-center retrospective cohort study included 201 patients with confirmed BSIs based on positive blood cultures. Data on patient characteristics, clinical outcomes, hospitalization costs, and laboratory parameters were analyzed using appropriate statistical methods. Primary BSIs occurred in 22.89% (46 patients), while secondary BSIs occurred in 77.11% (155 patients). Primary BSI patients were younger and predominantly nosocomial, whereas secondary BSI was mostly community-acquired. Clinical severity scores (SOFA, APACHE II, SAPS, and qPitt) were significantly higher in primary compared to secondary BSI. The median hospital stay was longer for primary BSI (21 vs. 12 days, *p* < 0.001). Although not statistically significant, mortality rates were higher in primary BSI (43.24% vs. 26.09%). Total care costs were significantly higher for primary BSI (EUR 4388.3 vs. EUR 2530.25, *p* = 0.016), driven by longer hospital stays and increased antibiotic costs. This study underscores the distinct clinical and economic challenges of primary versus secondary BSI and emphasizes the need for prompt diagnosis and tailored antimicrobial therapy. Further research should focus on developing specific management guidelines for primary BSI and exploring interventions to reduce BSI burden across healthcare settings.

## 1. Introduction

Bacteremia, a term synonymous with bloodstream infection (BSI), can either be primary in origin or a significant component of localized infections, i.e., secondary, and is associated with increased morbidity and mortality due to several clinical consequences [1]. Classifying bloodstream infections as primary or secondary is important for understanding the sources of infections and guiding treatment strategies. This classification helps determine the proportion of infections with no secondary cause defined, known as primary BSIs, and aids in identifying potential sources of infections, such as central line-associated BSIs (CLABSIs) [2], which may significantly impact patient outcomes and mortality rates [3]. Conversely, a source-confirmed secondary BSI should prompt early source control to aid in improving patient outcomes. Source control, defined as measures to eliminate the source of infection, is a key component in managing sepsis and septic shock, as it controls contamination and eliminates the infection source [4]. Delayed source control in bacteremia is associated with increased mortality rates [5]. A recent systematic review on the subject suggests that source control carried out within 6 h of evaluation in the emergency department significantly reduces short-term mortality compared to delayed intervention [6].

Furthermore, the classification of bloodstream infections as primary or secondary can impact the choice of antimicrobial therapy. Early, adequate antimicrobial therapy is crucial for improving patient outcomes, especially in cases of sepsis or septic shock [7]. The choice of first-line antimicrobials in healthcare-associated and hospital-acquired BSIs should consider local epidemiology, suspected sources, and documented colonization with multidrug-resistant (MDR) bacteria [8]. The type of pathogen causing a primary BSI can impact ICU mortality, with certain pathogens being associated with significantly higher mortality rates [9]. Only a few studies, however, have attempted to assess the mortality risk associated with the origin of the BSI or identify the most frequently isolated microorganisms [10,11,12]. Epidemiologically, distinguishing between primary and secondary bloodstream infections helps prioritize prevention efforts. For instance, a recent study concerning primary BSIs noted a high proportion of primary non-CLABSIs among hospital-acquired BSI events, suggesting the need for measures to prevent peripheral-line-associated BSIs [13]. Moreover, secondary BSIs, accounting for almost a quarter of all nosocomial bacteremia episodes [14], are poorly studied. Patients with secondary BSIs reportedly have multiple comorbidities, leading to increased hospital stays and mortality [14]. Therefore, measures to prevent the onset of secondary bacteremia, or at least diagnose it early, are of imminent importance.

To summarize, classifying bloodstream infections as primary or secondary is crucial for understanding sources of infections, guiding prevention strategies, and impacting treatment decisions, with significant clinical and epidemiological implications. It is finally important to note that BSI not only leads to significant clinical challenges but also imposes substantial economic burdens on healthcare systems [15]. The costs associated with BSI include prolonged hospital stays, increased and/or extended use of antibiotics, and additional diagnostic and therapeutic procedures [16]. In this study, we aimed to comprehensively investigate the differences in patterns, severity, outcomes, and medical costs between these two distinct clinical entities.

## 2. Materials and Methods

### 2.1. Study Design and Setting

This was a single-center, descriptive cross-sectional study with historical data collection, utilizing the registry of patients within the Department of Internal Medicine of the University Hospital of Patras, Greece, between May 2016 and May 2018. 

### 2.2. Study Population and Inclusion/Exclusion Criteria

The study population consisted of consecutive adult patients (age > 18 years) admitted to the hospital with a confirmed diagnosis of BSI based on a positive blood culture acquired upon admission. Patients were classified into primary or secondary BSI groups using the following criteria:

Primary BSI: Defined as a bloodstream infection without an identifiable source of infection after a thorough clinical examination and diagnostic testing, which included imaging studies (such as CT scans, MRIs, or ultrasounds) and microbiological cultures from potential primary sites of infection (such as urine, sputum, or wound cultures). If no source was identified through these diagnostic measures, the BSI was classified as primary.

Secondary BSI: Defined as a bloodstream infection with an identifiable source of infection at the time of positive blood culture. The identification of the source was confirmed through clinical assessments, imaging studies, and microbiological cultures. The common sources of secondary BSIs were categorized as follows:

- Urinary Tract Infections (UTIs): These were identified when patients presented with symptoms such as dysuria, frequency, urgency, or flank pain, along with positive urine cultures showing significant growth of uropathogens. Imaging studies (e.g., ultrasound or CT scan) could be used to identify structural abnormalities or complications such as abscesses.
- Intra-Abdominal Infections (IAIs): These were diagnosed in patients with abdominal pain, tenderness, or distension and were confirmed through imaging studies (such as CT scans) showing evidence of abscesses, appendicitis, diverticulitis, or other intra-abdominal pathologies. Microbiological cultures from aspirated fluids or tissues also supported the diagnosis.
- Lower Respiratory Tract Infections (LRTIs): Patients with LRTIs presented with symptoms such as cough, sputum production, dyspnea, or chest pain. The diagnosis was supported by chest imaging (such as X-rays or CT scans) showing infiltrates, consolidation, or abscesses. Sputum cultures or bronchoalveolar lavage fluid cultures provided microbiological confirmation.
- Skin and Soft Tissue Infections (SSTIs): These included cellulitis, abscesses, and wound infections. Patients presented with local signs of infection, such as erythema, warmth, swelling, and pain. The diagnosis was confirmed through clinical examination and, if necessary, imaging studies. Cultures from wound swabs or aspirated pus were used for microbiological confirmation.
- Other Infections: This category included less common sources of secondary BSIs, such as central nervous system infections, bone and joint infections, and endocarditis. These infections were identified through specific clinical presentations (e.g., neurological symptoms, joint pain) and confirmed by relevant imaging studies and microbiological cultures (e.g., cerebrospinal fluid cultures, joint aspirates, echocardiography for endocarditis).

Patients were excluded in the presence of evidence of blood culture contamination. Blood culture contamination was defined as the growth of specific environmental commensal organisms cultivated from a single blood culture, taken out of a blood culture series, that do not represent true bacteremia, e.g., *S. epidermidis*.

### 2.3. Data Collection and Clinical Outcomes

Data were extracted from the hospital’s electronic medical records system and patients’ records, including demographic data, clinical presentation, laboratory results, and treatment details. Outcome-related information, including all-cause mortality during hospitalization, and length of hospital stay (LOS), was also documented. Each patient record was reviewed to classify the BSI as primary or secondary based on the presence or absence of a localized infection source. The source of infection was determined through clinical assessments, imaging, and microbiological cultures. The severity scoring systems (APACHE II, SAPS II, SOFA, and qPitt score) were assessed when a positive blood culture was detected. Sepsis stage was assessed using Sepsis 2 and Sepsis 3 definitions [17,18]. 

### 2.4. Hospitalization Costs

Hospitalization costs were calculated according to the database of the Ministry of Health and the local administrative office, which is based on each ICD-10 code according to days of hospitalization [19]. Unit costs of antibiotic regimens were retrieved from the national formulary of the National Organization for Pharmaceuticals (EOF) [20] and then manually calculated by daily dosage, unit price, and length of stay for each patient.

### 2.5. Study Ethics

The study was conducted in accordance with the ethical standards of the 1964 Declaration of Helsinki. Approval was obtained from the local Ethics Committee and Institutional Review Board (96/15-04-2016).

### 2.6. Statistical Analysis

Categorical variables are presented as counts and relative frequencies, while continuous variables are presented as medians and interquartile ranges. Pearson’s chi-squared test, with Yates’ correction for continuity where possible, was used to assess associations between categorical variables. In cases where the data did not have the appropriate structure to use Pearson’s chi-squared test, Fisher’s exact test was used. The Shapiro–Wilk and Kolmogorov–Smirnov tests were used to test the normality of continuous variables. In minimal cases of discrepancy between the two tests, the result of the Shapiro–Wilk test was taken into account when there were less than 50 values in the sample, and the Kolmogorov–Smirnov test was taken into account when there were at least 50 values in the sample [21]. Continuous normally distributed variables were compared using the student’s t-test, and the Mann–Whitney U test was used to compare nonparametric variables. In the logistic regression model for the multivariate evaluation of the influence of each independent variable, the “Hosmer-Lemeshow” test was used to evaluate the goodness-of-fit of the model. The selection of variables included in the model was based on clinical relevance and statistical significance in the univariate analysis. With respect to the multiple linear regression model, multicollinearity was assessed using the Variance Inflation Factor (VIF) and a VIF < 2 was confirmed for all variables included. The “Durbin-Watson statistic” test was used to detect the presence of autocorrelation, and the variables were also based on clinical relevance. All results were followed by 95% confidence intervals (95% CI). All tests were two-tailed, and the level of statistical significance was set at *p* = 0.05. All analyses and graph generation were performed using RStudio 2023.06.1—Build 524 ( PBC, Boston, MA) with the following packages: “dplyr” v. 1.1.4, “ggplot2” v. 3.5.0, “scales” v. 1.3.0, “nortest” v. 1.0-4, “epiDisplay” v. 3.5.0.2, “broom” v. 1.0.5, “lmtest” v. 0.9-40, “car” v. 3.1-2, and “stats” v. 4.3.1.

## 3. Results

### 3.1. Patient Characteristics

A total of 201 patients with established BSI were ultimately included in this study. Among them, 22.8% (n = 46) developed primary BSI, while 77.1% (n = 155) developed secondary BSI (Table 1). Patients with primary BSI tended to be younger than those with secondary BSI. A significant proportion of primary BSI cases were nosocomial in origin, in contrast to the predominantly community-acquired nature of secondary BSI. In terms of origin in secondary BSI, UTIs predominated (33.6%), followed by IAIs (32.8%), lower respiratory tract infections (LRTI) (16.1%), SSTIs (9.7%), and other infections (7.8%). The stages of sepsis did not differ significantly between the bacteremia subgroups. The Charlson Comorbidity Index (CCI) scores were similar between the groups.

### 3.2. Isolated Pathogens

**Figure 1:**
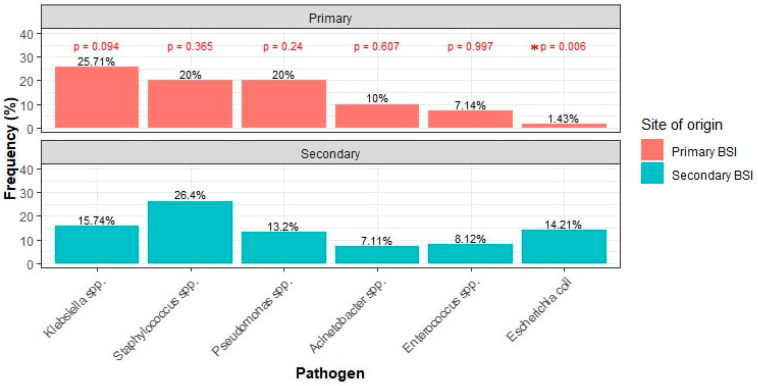
Most common pathogens between primary and secondary BSI. (* denotes statistical significance).

The number of isolated pathogens did not significantly differ between primary and secondary BSIs (Table 1). Both types of BSIs were predominantly monomicrobial. The distribution of pathogens by Gram staining was similar between the groups, with Gram-negative bacteria being the most common, followed by Gram-positive bacteria, and mixed/polymicrobial BSIs also involving fungi. The frequencies of isolates were compared across the two groups. The only statistically significant difference was the higher frequency of *Escherichia coli* in secondary bacteremia compared to primary bacteremia (Figure 1).

### 3.3. Laboratory Parameters and Prognostic Scores

Patients with primary BSI exhibited significantly higher white blood cell (WBC) counts and CRP levels and lower platelet counts and hemoglobin levels compared to those with secondary BSI (Table 1). In terms of clinical severity comparison between the groups, primary bacteremia patients had higher scores on the APACHE, SOFA, and SAPS scales (Table 1). Additionally, a higher percentage of primary bacteremia cases had a qPitt score ≥2 compared to secondary cases (Table 1).

### 3.4. Clinical and Economic Outcomes

**Figure 2:**
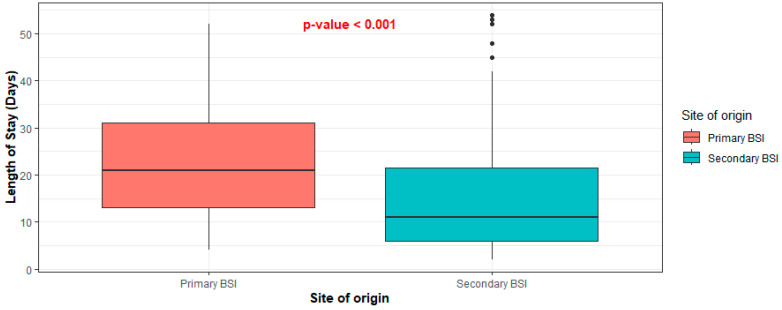
Distribution of length of stay between primary and secondary BSI.

**Figure 3:**
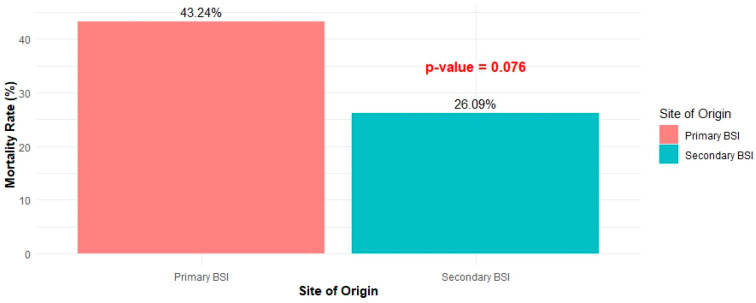
Mortality rate between primary and secondary BSI.

Cases diagnosed with primary BSI tended to be hospitalized longer than those diagnosed with secondary BSI. The median length of hospital stay (LOS) of the first group of patients was 21 days (IQR: 13–31), while that of the second group was 12 days (IQR: 6.5–23) (*p* < 0.001) (Figure 2). Finally, 43.24% of patients with primary bacteremia succumbed to disease, compared to 26.09% of patients with secondary bacteremia (*p* = 0.076) (Figure 3).

**Figure 4:**
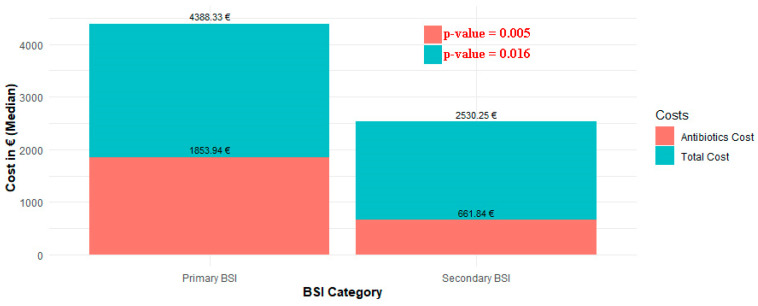
Distribution of total costs and antibiotic costs between primary and secondary BSI.

Both the cost of antibiotics and the total cost of care were found to differ significantly between cases of primary and secondary BSI (*p* = 0.005 and *p* = 0.016, respectively). Specifically, the median cost of antibiotics administered to patients with primary BSI was EUR 1853.9 (IQR: 594.4–6115.6) and the median total care cost was EUR 4,388.3 (IQR: 2421–7970.8), while the corresponding costs for patients with secondary BSI were EUR 661.84 (IQR: 222–1656.48) and EUR 2530.25 (IQR: 1190–4991), respectively (Figure 4).

### 3.5. Primary BSI and Mortality Risk

The type of BSI, age, CCI, the presence of qPitt ≥2, and the presence of severe sepsis/septic shock were analyzed for their association with the outcome of mortality. Univariate analysis showed that the CCI (OR = 1.19, 95% CI: 1.05–1.36, *p* = 0.009), qPitt ≥ 2 (OR = 10.96, 95% CI: 4.07–31.73, *p* < 0.001), and the presence of severe sepsis/septic shock (OR = 17.14, 95% CI: 7.21–46.19, *p* < 0.001) significantly influenced the odds of death. Additionally, the presence of primary BSI was not significantly associated with mortality, albeit with marginal statistical non-significance (OR = 2.16, 95% CI: 0.99–4.68, *p* = 0.05). On the other hand, age was not found to be a significant predictor of mortality. In multivariate analysis, only qPitt ≥ 2 (OR = 4.02, 95% CI: 1.09–15.52, *p* = 0.037) and the presence of severe sepsis/septic shock (OR = 7.42, 95% CI: 2.19–27.84, *p* < 0.001) remained statistically significant predictors. No statistical significance was found for the type of bacteremia or other variables. The results are presented in Table 2 for both univariate and multivariate analyses.

### 3.6. Healthcare Costs Associated with Primary Bacteremia

Furthermore, LOS and type of BSI were found to be statistically significant predictors of the total care cost (*p* = 0.002 and *p* = 0.029, respectively) using a multiple linear regression model for total care costs, adjusted for qPitt score ≥ 2, and CCI. Specifically, primary BSI resulted in an increase of EUR 6979.94 in total costs compared to secondary BSI, provided that the other 3 variables in the model are held constant. The multiple linear regression model is presented in Table 3.

## 4. Discussion

This study aimed to compare primary and secondary BSI characteristics across a wide variety of clinical-pertinent factors. Primary BSI patients tended to have a worse prognosis and result in higher healthcare costs, while secondary bacteremia was more frequently associated with community-acquired infections and exhibited higher inflammatory markers.

Patients with primary bacteremia were younger than those with secondary bacteremia, which aligns with previous findings indicating that younger patients might be more susceptible to primary bacteremia. One could argue that this is possibly due to fewer comorbid conditions in conjunction with less exposure to healthcare-associated risks that typically increase with age. However, the CCI reflecting the comorbidity burden was similar across the groups, and the setting of origin for primary bacteremia was predominantly the hospital. On the contrary, secondary bacteremia was more frequently community-acquired, indicating that infections at other body sites often transition into bloodstream infections, particularly in outpatient settings. Our findings align with those from a comprehensive study from Thailand assessing resistance patterns in bacteremias, which additionally reported on the differences between primary and secondary bacteremias [22]. The age of the patients with primary bacteremia was indeed younger (mean age 51.7 ± 28.8 years) compared to those with secondary bacteremia (mean age 61.9 ± 19.5 years) (*p* < 0.001). Primary bacteremia was also predominantly nosocomial (111/237; 46.8%), whereas secondary bacteremia was more frequently community-acquired (295/465; 63.4%) (*p* = 0.004). Taken together, these primary findings highlight the importance of stringent infection control measures in hospitals to prevent healthcare-associated primary BSIs, as well as proactive management of community-acquired infections to prevent secondary bacteremia [23]. 

Our study also reveals intriguing differences in hematological laboratory values and inflammatory markers. Patients with secondary BSI had significantly higher median WBC counts and CRP levels compared to those with primary BSI, indicating a more intense systemic inflammatory response in secondary bacteremia, likely due to underlying hollow viscous or deep-seated infections originating from specific body sites. While secondary BSI appears to provoke a stronger acute inflammatory response, primary BSI is associated with significant hematological alterations, reflecting potential different underlying mechanisms. The platelet count (PLT) was significantly lower in primary bacteremia, similar to hemoglobin levels. These findings suggest that primary bacteremia might be associated with more profound impacts on the hematological system, potentially indicating more extensive bone marrow suppression, possible hemolysis, and/or consumption coagulopathy [24]. Hematological values and corresponding indices, especially those involving neutrophils, have been evaluated for their prognostic value in various types of bacteremia. For example, the blood neutrophil-lymphocyte ratio has been associated with 90-day all-cause mortality in patients with bacteremia from Gram-negative pathogens [25]. However, their limited specificity precludes their clinical use and widespread implementation [26].

Complementing these findings, our study also found that primary BSI was associated with higher prognostic severity scores (SOFA, APACHE II, SAPS, and qPitt) in line with the similar trend in mortality rates. This finding suggests the utility and applicability of these severity scores in clinical practice for assessing the potential for severe outcomes in bacteremia patients. The widely used bacteremia-specific qPitt tool has proven its applicability in numerous scenarios of bacteremia involving various causative pathogens, both Gram-positive and Gram-negative [27,28]. In fact, qPitt has even been investigated for its prognostic value in non-bacteremic infections with much success [29]. On the other hand, clinical presentation in terms of the septic cascade as defined by the current international criteria (SEPSIS-3) [18] was not significantly different across the two groups. This raises concerns about the applicability of the Sepsis-3 criteria to the unique presentation of bacteremia patients, particularly regarding the early recognition and treatment of infections before organ dysfunction ensues [30]. 

In terms of causative pathogen epidemiology, our study identified a significant difference in the prevalence of *E. coli*, which was more common in secondary BSI (14.21% vs. 1.43%, *p* = 0.006). This pathogen predominance is typical in secondary BSI, where infections originate from specific body sites like the urinary tract, as also shown in our study [31]. Epidemiologically, primary BSIs in the ICU setting are often similar to nosocomial bacteremias and are predominantly caused by Gram-positive organisms such as coagulase-negative *staphylococci* and *S. aureus*, especially when associated with intravascular catheters or other implanted prosthetic materials [32]. Nosocomial Gram-negative pathogens like *Klebsiella*, *Pseudomonas*, and *Acinetobacter* are also common in these settings. In contrast, community-onset bacteremias among patients admitted to the ICU commonly involve *E. coli*, *S. aureus*, and *S. pneumoniae* [33]. Comparing our results, in terms of isolated bacteria, to the comprehensive study from Thailand discussed before [22], *E. coli* was also more common in secondary BSI (149/465; 32.0%) compared to primary BSI, although not as overwhelmingly as in our results (48/237; 20.3%) (*p* = 0.001). Moreover, *K. pneumoniae* was more frequently isolated in primary BSI (50/237; 21.1%) than in secondary BSI (61/465; 13.1%) (*p* = 0.005), in concordance with our findings. *P. aeruginosa* and *S. aureus* did not display significant differences across groups. On the other hand, *A. baumannii* was more common in secondary BSI (15/465; 9.7%) than primary BSI (11/237; 4.6%) (*p* = 0.02), possibly reflecting local epidemiology.

The higher mortality rate observed in primary BSI (43.24% vs. 26.09%), although not statistically significantly higher, highlights the severe potential of primary BSI per se and the difficulty in clinical management and treatment, potentially due to the challenge of early identification and the delay in administering effective antibiotics. The study from Thailand also reported that patients with primary bacteremia had a higher rate of non-concordant antibiotic therapy (49/228; 21.5%) compared to those with secondary BSI (139/444; 31.3%) (*p* = 0.007) [22]. Clinical response at the end of treatment was reported in 125/204 (61.3%) of primary BSI cases and 281/444 (63.3%) of secondary BSI cases (*p* = 0.59). The in-hospital mortality rate was 70/204 (34.3%) for primary BSI and 148/444 (33.3%) for secondary BSI (*p* = 0.81). Our study did not find primary BSI to be an independent predictor of mortality, which aligns with the understanding that, while primary BSI is associated with severe clinical outcomes, effective management can mitigate the risk of death. However, the presence of severe sepsis or septic shock and a qPitt score greater than 2 points were significant predictors of mortality, reinforcing the critical role of early and aggressive treatment in improving patient outcomes. It is important to note that our study analyzed only cases from the medical ward and not ICU cases. Other studies in ICU settings report conflicting findings concerning mortality outcomes, likely due to the heterogeneity of patient populations and their severity, as well as the epidemiology of the pathogens. For instance, a comprehensive study of 15 ICUs from France reported significantly higher mortality rates for secondary-nosocomial BSIs (55%) compared to primary and catheter-related BSIs (20%), with an odds ratio of 4.6 (95% CI 2.9–7.1) for mortality in secondary BSIs [12]. On the other hand, a cohort of 327 critically ill patients with sepsis from LRTI, IAI, and primary BSI origins found that primary BSI was associated with a significantly higher 90-day mortality risk compared to LRTI and IAI (HR 2.10; 95% CI 1.14 to 3.86; *p* = 0.0166) [11]. Specifically, the mortality rates were 58% for primary BSIs, 35% for LRTIs, and 32% for IAIs (*p* = 0.0208).

In the context of multidrug-resistant (MDR) pathogens, the origin of BSI as a mortality predictor is also debated. A recent study from Greece, which reports a high overall mortality rate of 50.3% in MDR-caused BSIs, indicated in their univariate analysis that the type of bacteremia (primary vs. secondary) did not significantly impact mortality (OR 1.21, 95% CI: 0.64–2.29, *p* = 0.560), suggesting that the source of BSI may not be the primary determinant of mortality in the context of MDR infections [34]. In another recent study from Colombia, which examined mortality in BSI due to extended spectrum β-lactamase (ESβL)-producing *Enterobacterales* with a hospital mortality rate of 38%, it was reported that there were no differences in mortality due to sepsis regarding the source of infection (primary bacteremia, secondary bacteremia, or catheter-related BSI) [35]. Significant risk factors for mortality included shock requiring vasoactive support, a Pitt score greater than 3 points, both of which were also reported as risk factors in our study—with the note that the cutoff for qPitt in our case was set at 2, based on previous studies indicating that it is a reasonable cut-off for critical disease [36,37]—and the absence of an infectious disease (ID) consultation. In our study, we had active and relatively early ID consultation based on hospital protocols, which is noteworthy as, based on findings from a retrospective population-wide cohort study concerning BSI, the absence of an ID consultation was associated with increased mortality [38]. Furthermore, ID consultation has been demonstrated to be cost-effective. For example, in the management of *S. aureus* bacteremia, ID consultation was associated with estimated savings of USD 55,613.4 per death averted and was cost-effective in 54% of simulations at a willingness-to-pay threshold of USD 50,000 [39].

In this respect, our study demonstrates that primary BSI incurs significantly higher healthcare costs compared to secondary bacteremia. Total care costs for primary bacteremia were significantly higher. This is not surprising, as managing infections without a clear source necessitates longer investigations, specialized interventions, and possibly extended treatment durations. A notable factor contributing to these elevated costs is the longer median LOS for primary BSI patients, which was 21 days versus 12 days for secondary BSI patients. Primary BSIs have been reported to increase LOS by an average of 7.4 days and increase charges by USD 3517 per episode in a Centers for Disease Control (CDC) study from 1992 [40]. According to our linear regression model, each additional day of hospitalization adds approximately EUR 266.4 to the total cost. Other factors contributing to the increased total cost, although intuitive and not previously reported, include elevated antibiotic therapy expenses and greater clinical severity, a fact clearly disclosed by our study.

The clinical severity of primary BSI cases also drives up costs. All severity indices calculated on the first day of hospitalization were higher in the primary BSI group, indicating more complex and severe cases requiring extensive and costly medical management. Despite this, neither the qPitt score > 2 nor the CCI score reached statistical significance in predicting total costs within our linear model. After all, these scores are designed to predict mortality rates rather than costs, as demonstrated by their significance in the mortality prediction model. The linear regression model employed in our study emphasizes that the type of bacteremia is the most important determinant of total healthcare costs. Specifically, primary BSI was associated with an additional cost of EUR 6979.94) making it the most significant predictor of total care costs. This “umbrella” factor includes all contributors to the total costs analyzed above, such as antibiotic therapy expenses, clinical severity, and length of hospitalization, but also the predominance of nosocomial origin, which need to be considered to fully understand the compartmentalization of the economic burden of primary bacteremia. Our findings align with those of previous studies on nosocomial infections reporting increased costs in primary BSIs [41,42,43]. For instance, a pivotal study from Spain identified that hospitalization costs varied significantly across different sources of bacteremia [44]. The highest yearly incremental costs were reported for primary or unknown foci of bacteremia: EUR 26,082 for antimicrobial-susceptible bacteremia (MDSM) and EUR 29,186 for multidrug-resistant bacteremia (MDRM). For comparison, urinary tract-originated bacteremias resulted in costs of EUR 6786 (MDSM) and EUR 13,299 (MDRM), while bacteremias with a gastrointestinal tract source incurred costs of EUR 15,633 (MDSM) and EUR 10,663 (MDRM). Endovascular device-related infections presented incremental costs of EUR 12,002 (MDSM) and EUR 14,516 (MDRM), illustrating the substantial economic impact of primary bacteremia on healthcare systems.

This study has several limitations that must be acknowledged. First, being a retrospective single-center study, the findings may be influenced by the specific practices and protocols of our healthcare facility, which could limit the generalizability of our results to other settings. Second, the retrospective design of the study inherently limits control over data collection and completeness. The potential for misclassification of BSIs due to insufficient investigation or incomplete clinical data is a possibility to be noted. Also, no data on specific risk factors such as a weakened immune system, recent surgeries involving catheters, burns, or intravenous drug use were collected. Third, the relatively small sample size of the cohort restricted our ability to perform a more detailed multivariate analysis. Despite our efforts to account for potential confounders, there remains the possibility that some relevant clinical variables were not captured or adequately adjusted for in our analysis. Fourth, the lack of detailed data on antibiotic treatment and the resistance status of the causative microorganisms presents a significant limitation. Without this information, it is uncertain whether patients received optimal antibiotic therapy, which could directly impact patient outcomes, including mortality and length of stay (LOS). Fifth, prior use of oral antibiotics could have affected culture results, potentially leading to false negatives or delayed pathogen identification. Sixth, the absence of data on multidrug-resistant (MDR) organisms limits our ability to fully understand the influence of antimicrobial resistance on the clinical outcomes of bacteremia [45]. Additionally, information on microbiological response was not available, which prevents a comprehensive understanding of the infection dynamics and potential differences in outcomes. Seventh, the study did not assess variations in clinical and laboratory variables over the course of the disease. Most of the clinical and laboratory data were based on the initial measurements at the time of presentation, which might not reflect changes that occurred during the treatment period. This could affect the interpretation of the association between these variables and patient outcomes. Despite these limitations, our study provides valuable insights into the clinical outcomes associated with primary and secondary bacteremia. These real-world data on patient characteristics, costs, and outcomes contribute to the existing literature and can help guide future research and clinical practices aimed at improving the care of patients with bloodstream infections.

In conclusion, this study explores the distinct clinical and economic challenges associated with primary and secondary bacteremia. Our results emphasize the critical need for effective management strategies to improve patient outcomes and mitigate the economic burden of BSIs. Prompt and accurate diagnosis, coupled with tailored antimicrobial therapy, are essential to improving both parameters. Further research is necessary to develop specific guidelines for managing primary bacteremia and to explore interventions that can effectively reduce the burden of both primary and secondary bacteremia in various healthcare settings.

## Figures and Tables

**Table 1:** Patient characteristics.

Characteristic		Site of Origin		
		Primary BSI (n = 46)	Secondary BSI (n = 155)	*p*
Age (years)		61.5 (50.75–71)	75 (58–82)	<0.001
Gender (Male)		24 (52.17%)	86 (55.48%)	0.820
Source of Infection		45	142	<0.001
	Community-Acquired	6 (13.33%)	90 (63.38%)	
	Nosocomial	39 (86.67%)	52 (36.62%)	
Sepsis Stage		45	142	0.694
	Infection	1 (2.22%)	9 (6.34%)	
	Sepsis	21 (46.67%)	71 (50%)	
	Severe Sepsis	19 (42.22%)	53 (37.32%)	
	Septic Shock	4 (8.89%)	9 (6.34%)	
qSOFA		1 (1–2)	1 (0–2)	0.388
Charlson Comorbidity Index		7 (4–9)	6 (4–7)	0.075
Pathogen Identification n(%)				0.318
	Gram Positive	12 (26.1)	49 (31.6)	
	Gram Negative	25 (54.4)	85 (54.8)	
	Polymicrobial	9 (19.6)	21 (13.6)	
Laboratory Values				
WBC (K/μL)		6.55 (0.72–10.8)	12.12 (7.55–16.53)	<0.001
Hb (g/dL)		9.5 (8.5–10.8)	11.6 (10–13)	<0.001
PLT (K/μL)		154.0 (15.00–227.0)	204.0 (151.0–281.0)	0.001
CRP (mg/dL)		10.43 (5.71–17.87)	15.59 (7.21–23.05)	0.038
SGOT (U/L)		23.5 (16–35)	28.5 (19–57.5)	0.082
Alb (g/dL)		3.2 (2.8–3.6)	3.1 (2.7–3.6)	0.681
Ur (mg/dL)		41.5 (30.75–90)	45.5 (31.25–72.5)	0.927
Cr (mg/dL)		1 (0.8–1.55)	1 (0.9–1.6)	0.549
Prognostic Scores				
APACHE		20 (14–24)	13 (8–17)	<0.001
SAPS		48 (38–62)	31 (22–41)	0.005
SOFA		6 (5–10)	3 (1–5)	0.001
qPitt ≥ 2 (%)		47.4	18.5	0.014

Results are presented as: count (n), frequency (%) or median (interquartile range). PLT, platelet count; WBC, white blood cells; Hb, hemoglobin; CPR, C-reactive protein; SGOT, serum glutamic-oxaloacetic transaminase; Alb, serum albumin; Ur, blood urea; APACHE, Acute Physiology and Chronic Health Evaluation; SAPS, Simplified Acute Physiology Score; SOFA, Sequential Organ Failure Assessment.

**Table 2:** Univariate and multivariate analysis of factors correlated with mortality.

	Unadjusted			Adjusted		
	Odds Ratio	95% CI	*p*-Value	Odds Ratio	95% CI	*p*-Value
Type of Bacteremia (Primary)	2.16	0.99–4.68	0.05	3.85	0.79–20.35	0.098
Age	1.02	1.00–1.05	0.081	1.04	0.99–1.09	0.115
Charlson Comorbidity Index (CCI)	1.19	1.05–1.36	0.009	1.22	0.96–1.57	0.103
qPitt ≥ 2	10.96	4.07–31.73	<0.001	4.02	1.09–15.52	0.037
Severe Sepsis/Septic Shock	17.14	7.21–46.19	<0.001	7.42	2.19–27.84	0.001

**Table 3:** Multiple linear regression model for total care costs.

	Linear Regression Model	
	Β (95% Confidence Interval)	*p*-Value
Constant	−2600.62 (−8115.29385, 2914.0527)	-
LOS (Days)	266.40 (99.72016, 433.0751)	0.002
Type of Bacteremia (Primary)	6979.94 (692.56539, 13,267.3193)	0.029
qPitt ≥ 2 (Yes)	−4311.71 (−10,174.23795, 1550.8184)	0.147
Charlson Comorbidity Index (CCI)	515.61 (−296.79190, 1328.0108)	0.210

Abbrevations: LOS; Length of stay.

## Data Availability

Data can be made available upon reasonable request from the corresponding author.
